# A function for ataxia telangiectasia and Rad3-related (ATR) kinase in cytokinetic abscission

**DOI:** 10.1016/j.isci.2022.104536

**Published:** 2022-06-06

**Authors:** Janna Luessing, Chituru C. Okowa, Emer Brennan, Muriel Voisin, Noel F. Lowndes

**Affiliations:** 1 Genome Stability Laboratory, Centre for Chromosome Biology, Biochemistry & School of Natural Sciences, National University of Ireland Galway, Galway, Ireland

**Keywords:** Biological sciences, Molecular biology, Cell biology, Functional aspects of cell biology

## Abstract

Abscission, the final stage of cytokinesis, occurs when the cytoplasmic canal connecting two emerging daughter cells is severed either side of a large proteinaceous structure, the midbody. Here, we expand the functions of ATR to include a cell-cycle-specific role in abscission, which is required for genome stability. All previously characterized roles for ATR depend upon its recruitment to replication protein A (RPA)-coated single-stranded DNA (ssDNA). However, we establish that in each cell cycle ATR, as well as ATRIP, localize to the midbody specifically during late cytokinesis and independently of RPA or detectable ssDNA. Rather, midbody localization and ATR-dependent regulation of abscission requires the known abscission regulator-charged multivesicular body protein 4C (CHMP4C). Intriguingly, this regulation is also dependent upon the CDC7 kinase and the known ATR activator ETAA1. We propose that in addition to its known RPA-ssDNA-dependent functions, ATR has further functions in preventing premature abscission.

## Highlights

• ATR localises non-canonically to the midbody during late cytokinesis
• Absence of ATR function results in faster abscission and increased binucleates
• CDC7 kinase and the ESCRT protein, CHMP4C are required for ATR midbody localisation
• ATR functions upstream of known abscission regulators, CHMP4B and ANCHR

## Graphical abstract

**Figure:**
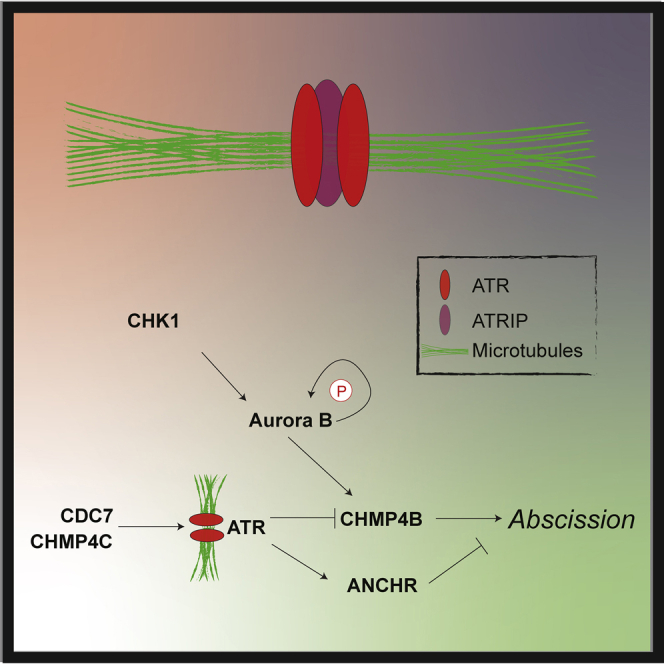

## Teaser

Luessing *et al* report a role for Ataxia Telangiectasia and Rad3-related (ATR) kinase in abscission independent of its RPA-ssDNA-dependent functions. They demonstrate that ATR negatively regulates abscission via the CDC7 kinase and CHMP4C.

## Introduction

Ataxia telangiectasia and Rad3-related (ATR), a phosphatidylinositol 3-kinase-related kinase (PIKK), is a central regulator of cellular responses to both replication stress and DNA damage (Yazinski and Zou, 2016; Saldivar et al., 2017; Blackford and Jackson, 2017). Critical to its role in these responses is its ability to interact with replication protein A (RPA)-coated single-stranded DNA (ssDNA) via its partner protein ATR-interacting protein (ATRIP). More recently, ATR has been implicated in Aurora B-dependent chromosome segregation (Kabeche et al., 2018; Bass and Cortez, 2019). Additionally, a role for ATR in a novel G2/M checkpoint has also been reported (Saldivar et al., 2018). While studying the S-M checkpoint using an Atr conditional null chicken DT40 cell line, we noticed that the absence of Atr led to an increase in binuclear cells (Eykelenboom et al., 2013), a phenotype often associated with defective cytokinesis (Norden et al., 2006; Steigemann et al., 2009).

In cytokinesis, immediately after segregation of the duplicated genome and downregulation of cyclin-dependent kinase (CDK1), a contractile ring of actin and myosin pinches the cytoplasmic membrane to form the cleavage furrow. This furrow ingression ultimately results in the formation of an intercellular bridge with a dense proteinaceous structure, the midbody, localized at its center upon overlapping plus-ended microtubules. Abscission is the last stage of cytokinesis in which the two daughter cells are separated and involves severing of microtubules and membrane fission of the cytoplasmic canal typically on either side of the midbody (Mierzwa and Gerlich, 2014; Horváth and Müller-Reichert, 2020; Peterman and Prekeris, 2019; D'Avino and Capalbo, 2016).

The final separation of daughter cells requires ESCRT (endosomal protein complex required for transport) complexes, in particular, interlocked ESCRT-III filaments (McCullough et al., 2018; Pfitzner et al., 2021). ESCRT-III filaments catalyze membrane remodeling, including fission of membrane necks from their luminal side, through complex exchanges of eight ESCRT-III proteins, all sharing a core structure and termed CHMP1 to 8 (charged multivesicular body proteins). Adding further complexity, there are two isoforms of CHMP1 (CHMP1A/B) and CHMP2 (CHMP2A/B), with three isoforms of CHMP4 (CHMP4A/B/C). Conceptually, within ESCRT-III filaments, the specific composition of CHMP proteins and their membrane-binding interfaces generates unique membrane shapes. ESCRT-III filaments are also highly dynamic with continuous remodeling being achieved by vacuolar protein sorting-associated protein 4 (VPS4), an AAA ATPase which forms a hexameric open ring that can pull individual CHMP subunits out of ESCRT-III filaments, denaturing them as it does so (Thoresen et al., 2014).The emerging model for ESCRT-III function during abscission suggests that the initially templated ESCRT-III filament recruits a secondary filament that is energetically unfavorable thereby storing elastic energy that is released upon VPS4-mediated disruption of the initial ESCRT-III filament to effect membrane constriction and eventual fission.

Here, we reveal a role for ATR in cytokinesis, specifically regulation of abscission. ATR, as well as its partner, ATRIP, are recruited to the midbody of the cytoplasmic canal during late cytokinesis and negatively regulate abscission. This recruitment is independent of RPA, TOPBP1, and ssDNA, as well as CHK1, ATM, and DNA-PK. Mechanistically, we establish that ATR midbody recruitment is dependent upon ATRIP and ETAA1, as well as CDC7 kinase activity and the CHMP4C isoform, which is known to negatively regulate abscission. ATR kinase activity is required with CHMP4C to impede the recruitment of CHMP4B isoform, the major positive regulator of abscission, as well as recruiting, ANCHR, another negative regulator of abscission, to the midbody.

## Results

### ATR localizes to the midbody in late cytokinetic cells together with RPA and ATRIP

**Figure 1:**
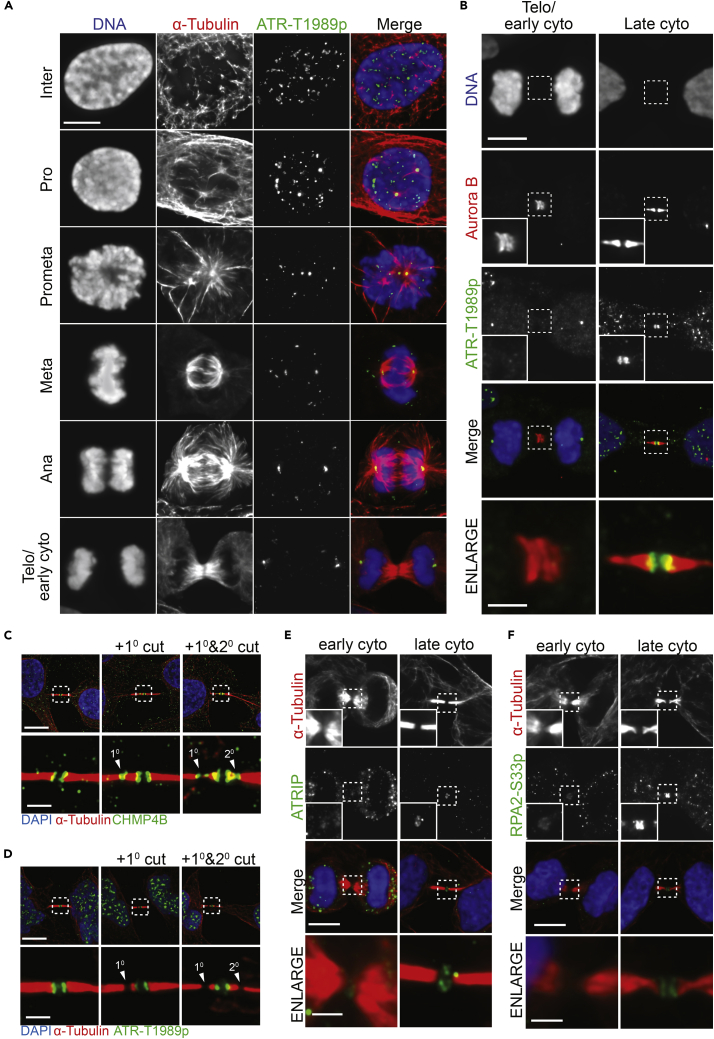
ATR localizes to the midbody in late cytokinesis independent (A) Cell-cycle-dependent localization of ATR-T1989p. (B) Cytokinetic localization of ATR-T1989p (green) and Aurora B (red). Note that in early cytokinesis, DNA condensation is still present, whereas in late cytokinesis DNA is decondensed. (C) Localization of CHMP4B in late cytokinesis and during abscission. The primary and secondary cut sites are indicated by white arrowheads. (D) Localization of ATR-T1989p in late cytokinesis and during abscission. The primary and secondary cut sites are indicated by white arrowheads. (E) Localization of ATRIP to midbodies in early and late cytokinetic cells. (F) Localization of RPA2-S33p to midbodies in early and late cytokinetic cells. All scale bars are 10 μm, all enlarged image scale bars are 2 μm. See also Figure S1.

The midbody is a signaling hub including known regulators of abscission (D'Avino and Capalbo, 2016; Peterman and Prekeris, 2019). We examined ATR localization during cytokinesis in HeLa and hTERT-RPE1 cells (Figures 1A, 1B, and S1A) using an antibody specific for ATR phosphorylation at Thr1989 (ATR-T1989p), a marker specific for active ATR (Nam et al., 2011). Immunofluorescence and western analyses confirms the specificity of this marker (Figures S1B and S1C). Additionally, we note that ATR-T1989p is detected specifically at nuclear speckles during interphase but not at sites of active transcription or at centromeres (Figures S1D–S1G). However, this marker is re-localized during mitotic prophase to centrosomes until late cytokinesis (Figures 1A, S1A, and S1H). Unlike Aurora B, which is at the midbody throughout cytokinesis, ATR was detected at the midbody only during late cytokinesis (Figure 1B).

Of the eight families of related charged multivesicular body proteins (CHMP1-7 and CHMP8/IST1) that form ESCRT-III filaments, CHMP4B is critically required for abscission (Elia et al., 2011, 2012; Guizetti et al., 2011). In contrast, its isoform, CHMP4C, is a negative regulator of abscission (Carlton et al., 2012; Thoresen et al., 2014). Consistent with its critical role in abscission, CHMP4B is recruited to the midbody in late cytokinesis and subsequently re-localizes to the primary and secondary abscission sites, which appear as narrow constriction zones with reduced or absent α-tubulin staining (Figure 1C). However, neither the primary nor secondary abscission sites displayed detectable ATR-T1989p (Figure 1D), suggesting that ATR is not required for the final fission of the cytoplasmic membrane as it is retained at the midbody during abscission.

Canonical ATR recruitment and activation at ssDNA is well known to be dependent upon association with ATRIP and RPA (Ball et al., 2005; Zou and Elledge, 2003)*.* We were unable to localize non-phosphorylated ATR to midbodies for technical reasons. However, consistent with the ATR-ATRIP heterotetrameric structure (Rao et al., 2018), ATRIP also follows the same localization pattern as ATR, being absent from early cytokinetic cells but localized to the midbody during late cytokinesis (Figure 1E). RPA2-S33p, an ATR-dependent phospho-form of RPA2 (Olson et al., 2006), as well as RPA1, were both detected at the midbody of all cells in late cytokinesis independently of DNA (Figures 1F and S1I). Thus, ATR, ATRIP, and RPA are each recruited to midbodies during each cell cycle.

### ATR localizes to the midbody in late cytokinetic cells independently of DNA, TOPBP1, and RPA but requires ATRIP and ETAA1

**Figure 2:**
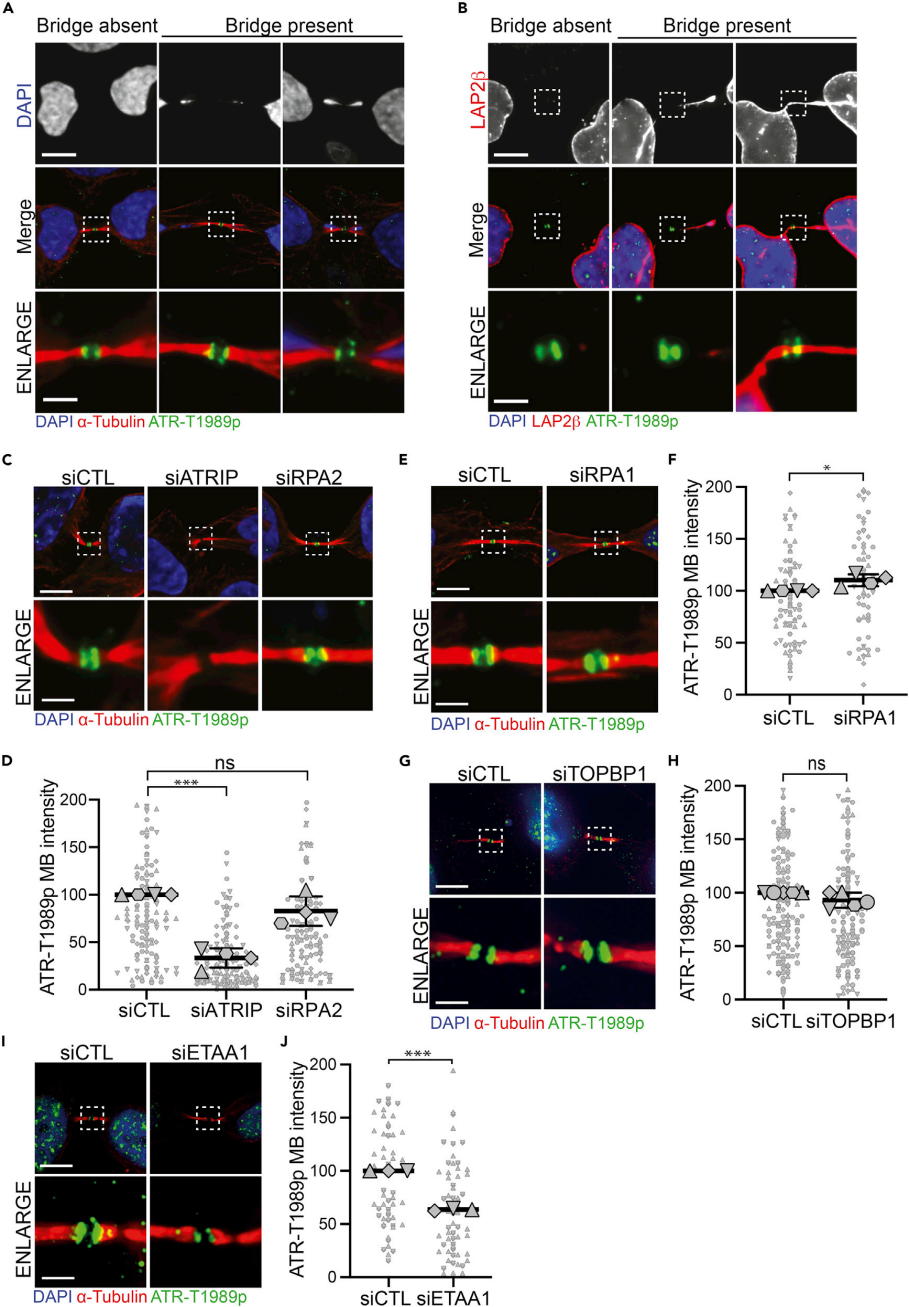
ATR localization to the midbody is dependent on ATRIP and ETAA1 but independent of DNA, RPA, and TOPBP1 (A) ATR-T1989p localizes to midbodies in all cytokinetic cells irrespective of the presence or absence of DAPI bridges. (B) ATR-T1989p localizes to midbodies in all cytokinetic cells irrespective of the presence or absence of LAP2β bridges. (C) Localization of ATR-T1989p to late cytokinetic midbodies upon depletion of ATRIP or RPA2. (D) Quantification of ATR-T1989p intensity at late cytokinetic midbodies upon depletion of ATRIP or RPA2 (*∗∗∗*p = *0.001, ns*; Student’s t-test). (E) Localization of ATR-T1989p to late cytokinetic midbodies upon depletion of RPA1. (F) Quantification of ATR-T1989p intensity at late cytokinetic midbodies upon depletion of RPA1 (*∗*p = *0.0382*; Student’s t-test). (G) Localization of ATR-T1989p to late cytokinetic midbodies upon depletion of TOPBP1. (H) Quantification of ATR-T1989p intensity at late cytokinetic midbodies upon depletion of TOPBP1 (ns; Student’s t-test). (I) Localization of ATR-T1989p to late cytokinetic midbodies upon depletion of ETAA1. (J) Quantification of ATR-T1989p intensity at late cytokinetic midbodies upon depletion of ETAA1 (∗∗*∗*p = *0.0004*; Student’s t-test). All dot plots represent data from at least three independent biological replicates, each replicate is represented by a different symbol. All data have been normalized to the control sample. Data are represented as mean ± SD of the individual replicates. HeLa cells were used in A–J. All representative images shown were selected from one of at least three independent experiments and stained as indicated. All scale bars are 10 μm, all enlarged image scale bars are 2 μm. See also Figure S2.

In response to replication stress, ATR is recruited to ssDNA (Zou and Elledge, 2003; Ball et al., 2005). Therefore, we examined the localization of ATR-T1989p to the midbody of cytokinetic cells in the presence or absence of detectable DNA or LAP2β bridges (Figures 2A and 2B, note that LAP2β is an inner nuclear envelope marker and indicates continuity of nucleoplasm across the cytoplasmic canal). Notably, ATR-T1989p was detectable at the midbody of all late cytokinetic cells examined, independently of whether their cytoplasmic canals contained detectable DNA or were positive for LAP2β. Similarly, ATRIP, as well as RPA2-S33p, also localized to midbodies of cytokinetic cells irrespective of the presence or absence of detectable DNA or LAP2β bridges (Figures S2A and S2B). Our observations suggest that ATR localization to the midbody is not facilitated by the presence of DNA trapped within the cytoplasmic canal; rather, it is an event that occurs in each and every cell cycle.

Next, we investigated whether recruitment of ATR to the midbody is regulated by proteins implicated in the function of ATR at stalled replication forks. We depleted its partner ATRIP, components of the RPA complex, required for its recruitment to stalled replication forks, or regulators of its activity, TOPBP1 or ETAA1. While ATR localization to the midbody was, unsurprisingly, dependent upon ATRIP and the consequent structural integrity of the ATR-ATRIP complex, neither RPA2 nor RPA1 was required (Figures 2C–2F and S2C–S2G). Thus, although RPA is present at midbodies during late cytokinesis, it is not required for ATR-ATRIP midbody localization. TOPBP1 is primarily responsible for activating ATR at stalled replication forks during S phase (Garcia et al., 2005; Jeon et al., 2011; Saldivar et al., 2017), with contributions from ETAA1 (Bass et al., 2016; Feng et al., 2016; Haahr et al., 2016; Lee et al., 2016). ETAA1 also has important roles in mitosis, where it is required for chromosome alignment and a fully functional spindle assembly checkpoint (Bass and Cortez, 2019). Depletion of TOPBP1 had no effect on ATR recruitment to the midbody (Figures 2G, 2H, and S2H), while depletion of ETAA1 moderately reduced localization of active ATR to the midbody of late cytokinetic cells (Figures 2I, 2J, and S2I). However, we cannot exclude the possibility that the reduction of ATR at the midbody upon ETAA1 depletion is an indirect effect due to ETAA1’s mitotic function. These data suggest that localization of ATR to the midbody of late cytokinetic cells is dependent upon its interacting partner protein ATRIP and ETAA1, but independent of TOPBP1 or the RPA complex, indicating distinct ATR regulation at midbodies relative to stalled replications forks.

### ATR negatively regulates abscission

**Figure 3:**
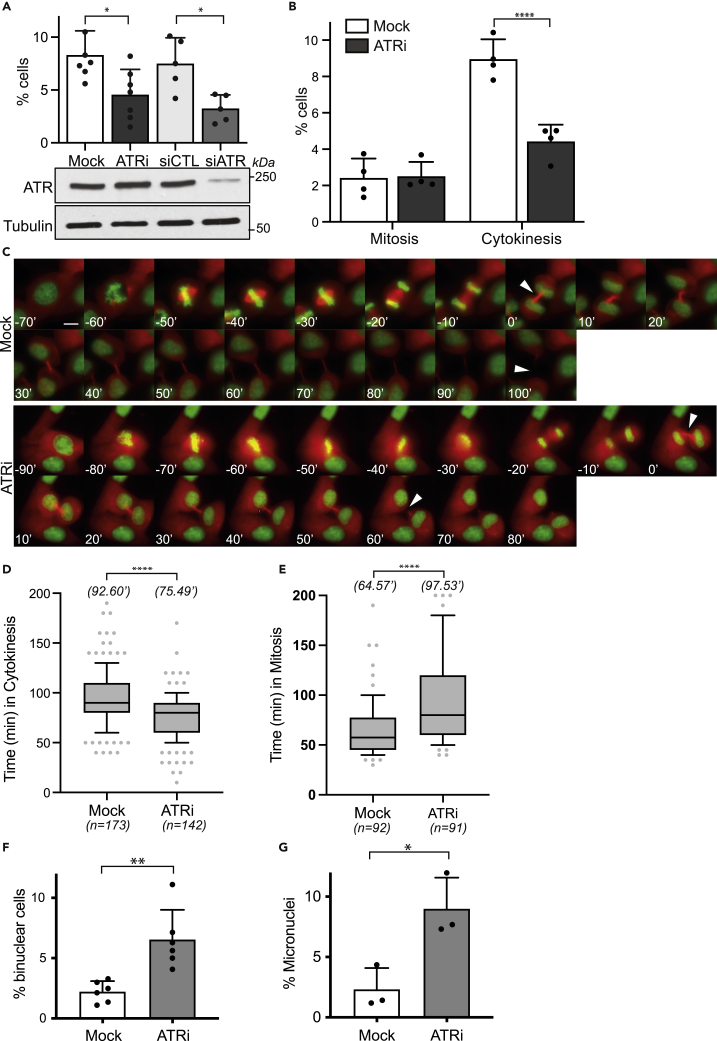
ATR is a negative regulator of abscission required for genome stability (A) Percentage of cytokinetic cells upon pharmacological inhibition (10 μM ETP-46464; 1 h) or genetic depletion of ATR. The Western blots indicate the expression levels of ATR and the α-tubulin loading control. At least, 200 cells were analyzed in each of a minimum of 5 independent experiments (∗p = 0.0214, ∗p = 0.0029; Student’s t-test). (B) Percentage of mitotic and cytokinetic cells upon pharmacological inhibition of ATR (ETP-46464, 10 μM; 1h). At least, 400 cells were analyzed in each of 4 independent experiments (∗∗∗∗p = 0.0001, Student’s t-test). (C) Selected frames of single cells from live cell microscopy using asynchronously growing HeLa cells stably expressing GFP-H2B and mCherry-α-tubulin. Cells were treated with DMSO (mock) or ATR inhibitor (ETP-46464, 10 μM) immediately prior to a 16 h time-lapse experiment. Mitosis was measured from visible chromosome condensation in prophase to separation of chromosomes in telophase. Abscission timing was determined by measuring time from a visible cytoplasmic canal separating daughter cells with decondensed chromatin to complete microtubule severance. (D) Quantification of abscission timing illustrated in (C). The number of cells analyzed across 3 independent experiments is indicated below each column (∗∗∗∗p = 0.0001, Student’s t-test). (E) Quantification of mitotic timing illustrated in (C). The number of cells analyzed across 3 independent experiments is indicated below each column (∗∗∗∗p = 0.0001, Student’s t-test). (F) Quantification of binuclear cells in response to ATR inhibition (ETP-46464, 10 μM) across six independent experiments (∗∗p = 0.0024, Student’s t-test). (G) Quantification of micronuclei in response to ATR inhibition (ETP-46464, 10 μM) across three independent experiments (∗p = 0.0210, Student’s t-test). Data are represented as mean ± SD of the individual replicates in panels A,B, F, and G indicate SD, boxplot of panels in E and F indicate the median and quartile ranges. HeLa cells were used in A–G. All scale bars are 10 μm. See also Figure S3.

To further investigate the role of ATR in cytokinesis, we analyzed asynchronously growing HeLa cells in which ATR was either pharmacologically inhibited or genetically depleted. Both approaches resulted in approximately 50% fewer cytokinetic cells (Figure 3A). Importantly, acute (1 h) inhibition of ATR did not interfere with mitotic progression but did significantly reduce the number of cytokinetic cells (Figures 3B and S3A).

We also examined the effect of ATR inhibition of cytokinesis using the NUP153 depletion assay, a proxy for the Aurora B-dependent abscission checkpoint (Mackay et al., 2010). In this assay, specific depletion of the nuclear pore component, NUP153, leads to an increased proportion of cytokinetic cells. Consistent with our detection of fewer cytokinetic cells upon ATR inhibition (Figure 3A), the increased proportion of cytokinetic cells resulting from NUP153 depletion was also reduced upon ATR inhibition (Figure S3B).

Additionally, live cell microscopy revealed significantly faster progression of ATR-inhibited cells through cytokinesis (Figures 3C and 3D). We also confirmed previous findings that ATR activity is required for mitotic progression (Bass and Cortez, 2019; Kabeche et al., 2018) as ATR inhibition prolonged mitosis significantly (Figure 3E). Prolonged exposure to ATR inhibitor may result in defective ATR function from earlier in the cell cycle causing mitotic delay compared to short acute treatments (Figure S3C). Nevertheless, both short and long ATR inhibition resulted in similar advancement of cytokinesis relative to control (Figure S3D), consistent with a distinct role for ATR in the regulation of cytokinesis. Both delayed mitosis and faster progression through cytokinesis resulted in significantly increased cell death (Figure S3E).

Thus, irrespective of its earlier functions in the cell cycle, both fixed and live cell data are consistent with ATR kinase activity being required for properly coordinated abscission. Importantly, and consistent with our previous report using chicken Atr conditional null DT40 cells (Eykelenboom et al., 2013), we confirmed that ATR inhibition in human cells also increases the frequency of both binuclear cells and micronuclei (Figures 3F and 3G), types of genome instability that have been linked to defective control of abscission (Steigemann et al., 2009; Petsalaki and Zachos, 2016). Our data support a role for ATR in regulating abscission that is important for maintenance of genome stability.

### ATR localization is independent of PIK and CHK1 kinases but dependent upon CDC7 kinase

**Figure 4:**
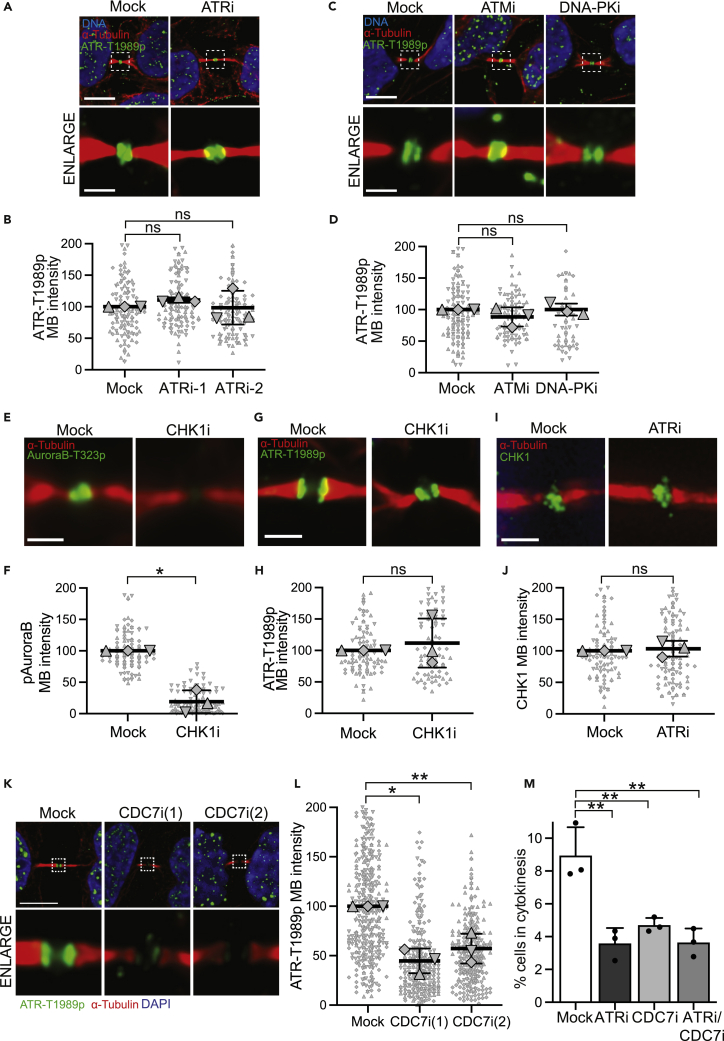
ATR-T1989p localization is independent of PIK and CHK1 kinase activities but dependent upon CDC7 (A) Localization of ATR-T1989p to the midbody of late cytokinetic cells upon ATR inhibition (ETP-46464, 10 μM; 1 h). (B) Quantification of ATR-T1989p intensity at the midbody of late cytokinetic cells upon ATR inhibition (ATRi-1 = ETP-46464, ATRi-2 = VE-821; 1 h) across three independent experiments (ns, Student’s t-test). (C) Localization of ATR-T1989p to the midbody of late cytokinetic cells upon ATM (KU55933, 10 μM; 1 h) or DNA-PK (NU7441,10 μM; 1 h) inhibition. (D) Quantification of ATR-T1989p intensity at the midbody of late cytokinetic cells upon ATM (KU55933, 10 μM; 1 h) or DNA-PK (NU7026,10 μM; 1 h) inhibition across three independent experiments (ns, Student’s t-test). (E) Localization of Aurora B-T323p to the midbody of late cytokinetic cells upon CHK1 inhibition (MK-8776, 10 μM; 1 h). (F) Quantification of Aurora B-T323p intensity at the midbody of late cytokinetic cells upon CHK1 (MK-8776, 10 μM; 1 h) inhibition across three independent experiments (∗p = 0.0162, Student’s t-test). (G) Localization of ATR-T1989p to the midbody of late cytokinetic cells upon CHK1 inhibition (MK-8776, 10 μM; 1 h). (H) Quantification of ATR-T1989p intensity at the midbody of late cytokinetic cells upon CHK1 inhibition (MK-8776, 10 μM; 1 h) across three independent experiments (ns, Student’s t-test). (I) Localization of CHK1 to the midbody of late cytokinetic cells upon ATR inhibition (ETP46464, 10 μM; 1 h). (J) Quantification of CHK1 intensity at the midbody of late cytokinetic cells upon ATR inhibition (ETP46464, 10 μM; 1 h) across three independent experiments (ns, Student’s t-test). (K) Localization of ATR-T1989p to the midbody of late cytokinetic cells upon CDC7 inhibition (CDC7i-1 = XL413, 10 μM, CDC7i-2 = PHA-767491, 10 μM; 1 h). (L) Quantification of ATR-T1989p intensity at the midbody of late cytokinetic cells upon CDC7 inhibition (CDC7i-1 = XL413, 10 μM; CDC7i-2 = PHA-767491, 10 μM; 1 h) across three independent experiments (∗p = 0.0165, ∗∗p = *0.0388;* Student’s t-test). (M) Percentage of cells in cytokinesis upon ATRi (ETP-46464, 10 μM; 1 h) and/or CDC7i (XL413, 10 μM; 1 h). At least, 400 cells were analyzed in each of 3 independent experiments (∗∗p = 0.0015, 0.0063, and 0.0016 for ATRi, CDC7i, and ATRi/CDC7i, respectively; Student’s t-test). All dot plots represent data from three independent biological replicates, each replicate is represented by a different symbol. All data have been normalized to the control sample. Data are represented as mean ± SD of the individual replicates. HeLa cells were used in A–M. See also Figure S4.

In the damage response, ATR is activated by ATR-dependent phosphorylation at Thr1989 (Nam et al., 2011). However, ATR-T1989p levels at the midbody did not change upon inhibition of ATR kinase activity (Figures 4A and 4B). Basal levels of ATR-T1989p were detected in untreated cells, as well as HU- and ATRi-treated cells (Figures S4A and S4B). The basal ATR-T1989p signal corresponds to phosphorylation as it is sensitive to lambda phosphatase treatment (Figure S4C), suggesting phosphorylation by another kinase. ATM and DNA-PK are related PIK kinases that have been reported to localize to midbodies (Yang et al., 2011; Douglas et al., 2014; Huang et al., 2014). However, neither of these PIK kinases had an effect on ATR-T1989p localization to the midbody (Figures 4C and 4D). Thus, ATR phosphorylation on Thr1989 and its localization to the midbody appears to involve a separate pathway that is independent of RPA and ATR-dependent phosphorylation of this residue. This phosphorylation and localization is also independent of ATM or DNA-PK.

During replication stress, the CHK1 kinase is a substrate of ATR (Bartek and Lukas, 2003). Additionally, CHK1 has a separate role in cytokinesis as an upstream regulator of Aurora B (Mackay and Ullman, 2015), a key regulator of abscission (Norden et al., 2006; Steigemann et al., 2009). However, while CHK1 inhibition reduced Aurora B intensity at the midbody (Figures 4E and 4F), it had no effect on ATR-T1989p localization (Figures 4G and 4H). Consistent with CHK1 functioning upstream of Aurora B in cytokinesis, inhibition of Aurora B did not alter ATR-T1989p localization (Figure S4D, upper panels, and E), despite the previously reported destabilization of microtubule bundles within the cytoplasmic canal upon Aurora B inhibition (Mallampalli et al., 2013). Additionally, although CHK1 is a substrate of ATR during replication stress, ATR inhibition had no effect on the localization of CHK1 to the midbody (Figures 4I and 4J). Thus, ATR and CHK1 have separable and distinct roles in cytokinesis.

Having ruled out roles for ATM, DNA-PK, CHK1, and Aurora B in localization of ATR-T1989p to the midbody, we next considered CDC7, a kinase normally associated with DNA replication (Rossbach et al., 2017). However, budding yeast Cdc7 has been shown to regulate mitotic exit (Miller et al., 2009). Additionally, human CDC7 has been reported to regulate mitotic progression, the spindle assembly checkpoint, and progression through cytokinesis (Nakuci et al., 2006; Ito et al., 2019). Acute inhibition of CDC7, using two chemically distinct inhibitors, significantly reduced the intensity of ATR-T1989p localization to midbodies without affecting its nuclear intensity (Figures 4K, 4L, and S4D, lower panels, to F). This suggests a function for CDC7 in regulating ATR localization to the midbody of cytokinetic cells. To define the relationship between CDC7 and ATR, we acutely inhibited either or both kinases and performed mitotic profiling. Acute treatment with either or both of these inhibitors had no effect upon cells in mitosis but significantly reduced the number of cytokinetic cells (Figures 4M, S4G, and S4H), consistent with both CDC7 and ATR functioning in the same pathway.

### ATR regulates abscission downstream of CHMP4C but upstream of ANCHR and CHMP4B

**Figure 5:**
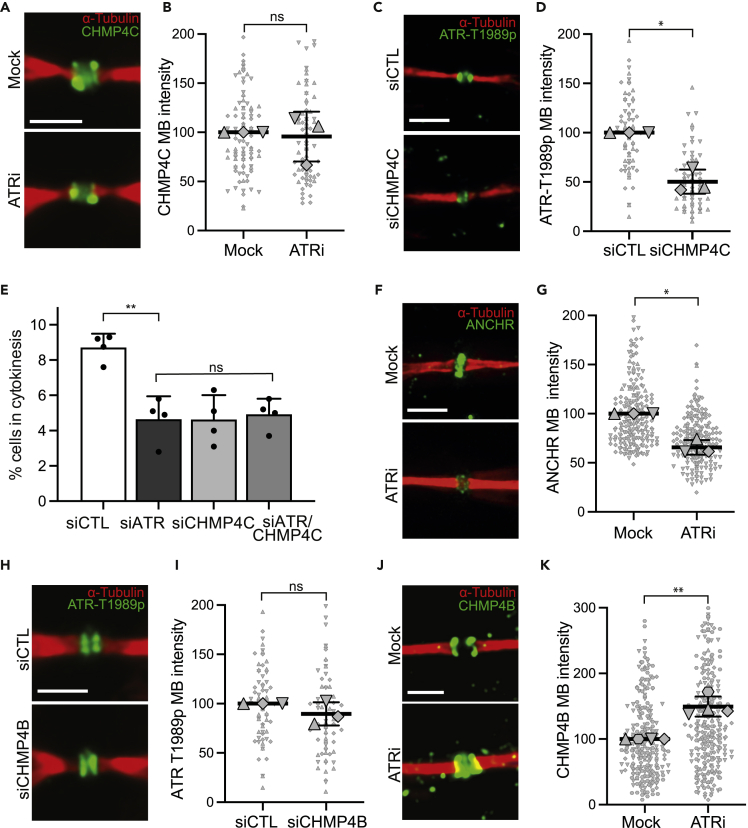
ATR functions downstream of CHMP4C but upstream of CHMP4B and ANCHR in the regulation of abscission (A) Localization of CHM4PC to the midbody of late cytokinetic cells upon ATR inhibition (ETP-46464, 10 μM; 1 h). (B) Quantification of CHMP4C intensity at the midbody of late cytokinetic cells upon ATR inhibition (ETP-46464, 10 μM; 1 h),(ns, Student’s t-test). (C) Localization of ATR-T1989p to late cytokinetic midbodies upon depletion of CHMP4C. (D) Quantification of ATR-T1989p intensity at late cytokinetic midbodies upon depletion of CHMP4C. (*∗*p = *0.0195*; Student’s t-test). (E) Percentage of cells in cytokinesis upon depletion of ATR and/or CHMP4C. At least, 400 cells were analyzed in each of 4 independent experiments (∗∗p = 0.0017; Student’s t-test). (F) Localization of ANCHR to late cytokinetic midbodies upon ATR inhibition (ETP-46464, 10 μM; 1 h). (G) Quantification of ANCHR intensity at late cytokinetic midbodies upon ATR inhibition (ETP-46464, 10 μM; 1 h). (*∗*p = *0.0153*; Student’s t-test). (H) Localization of ATR-T1989p to late cytokinetic midbodies upon depletion of CHMP4B. (I) Quantification of ATR-T1989p intensity at late cytokinetic midbodies upon depletion of CHMP4B. (ns; Student’s t-test). (J) Localization of CHM4B to the midbody of late cytokinetic cells upon ATR inhibition (ETP-46464, 10 μM; 1 h). (K) Quantification of CHM4B intensity at the midbody of late cytokinetic cells upon ATR inhibition (ETP-46464, 10 μM; 1 h). (∗*∗*p = *0.0077*; Student’s t-test). All dot plots represent data from three independent biological replicates, each replicate is represented by a different symbol. All data have been normalized to the control sample. Data are represented as mean ± SD of the individual replicates. All scale bars are 10 μm, all enlarged image scale bars are 2 μm. HeLa cells were used in A–K. See also Figure S5.

Abscission requires multiple components of the ESCRT-III complex (Elia et al., 2011). In particular, CHMP4C is a negative regulator, while the related CHMP4B is a positive regulator of abscission (Carlton et al., 2012; Thoresen et al., 2014). ANCHR is an additional negative regulator of abscission, reported to function during the Aurora B-dependent abscission checkpoint abscission that responds to chromatin in the cytoplasmic canal (Thoresen et al., 2014). Therefore, we assessed the relationship between ATR and these three abscission regulators. While ATR inhibition had no effect on CHMP4C localization to the midbody (Figures 5A and 5B), depletion of CHMP4C resulted in significant reduction of ATR-T1989p intensity (Figures 5C, 5D, and S5A). This suggests CHMP4C is required for ATR-T1989p localization to midbodies. Furthermore, depletion of either or both CHMP4C and ATR resulted in similarly reduced cytokinetic cells and this epistatic relationship supports functions for both these proteins in the same pathway (Figure 5E and see Figure S5E). With respect to the other negative regulator of abscission, ANCHR, inhibition of ATR resulted in significantly reduced recruitment of ANCHR to the midbody (Figures 5F and 5G), suggesting that ATR also functions upstream of ANCHR in the regulation of abscission. Moreover, we show that CDC7 inhibition as well as inhibition of both ATR and CDC7 resulted in a similar reduction of ANCHR levels at the midbody, suggesting that both of these kinases function epistatically (Figures S5B and S5C; see Figure S5E). On the other hand, depletion of CHMP4B had no effect upon this ATR-T1989p localization (Figures 5H, 5I, and S5D), while ATR inhibition increased the level of CHMP4B at midbodies (Figures 5J and 5K). Thus, both these negative regulators, CHMP4C and ATR, are required to impede recruitment of the CHMP4B positive regulator during normal cytokinesis, with CHMP4C functioning upstream, while ANCHR and CHMP4B function downstream, of ATR (see Figure S5E).

Here, we establish a role for ATR in the regulation of abscission timing. ATR is recruited to the midbody in a CDC7 and CHMP4C-dependent mechanism. At the midbody, ATR negatively regulates the localization of CHMP4B, a key positive regulator of abscission, while positively regulating the recruitment of ANCHR, a negative regulator of abscission.

## Discussion

ATR functions with ATRIP in a large heterotetrameric complex (Cortez et al., 2001; Rao et al., 2018). Here, we report that both components of this complex localize to the midbody specifically during late cytokinesis and that ATRIP is required for midbody localization of ATR. While the structural integrity of the ATR-ATRIP complex is likely important for ATR functions at the midbody, we cannot rule out a direct role for ATRIP in midbody recruitment of this complex. However, unlike ATR-ATRIP localization to RPA-coated ssDNA during replication stress and after DNA damage, its midbody localization is independent of RPA and detectable ssDNA. In fact, all late cytokinetic cells display ATR and ATRIP midbody localization irrespective of whether DNA or nucleoplasm can be detected within the cytoplasmic canal. Thus, ATR midbody localization is independent of an abscission checkpoint that responds to chromatin in the cytoplasmic canal and consistent with a role required in all cells as they transit through cytokinesis.

ATR regulates passage through cytokinesis as its inhibition or depletion results in fewer cytokinetic cells, while live cell microscopy revealed significantly faster progression of ATR-inhibited cells through cytokinesis. While the faster progression of ATR-inhibited cells through cytokinesis using live cell microscopy is significant, it is of relatively small effect size. However, small defects in cell cycle progression can result in dramatic phenotypes. For example, in mouse neuronal progenitor cells, a 10 min defect in transitioning mitosis activates the mitotic surveillance pathway resulting in TP53-dependent cell death and microcephaly (Phan et al., 2021), a phenotype that also occurs in ATR hypomorphic Seckel syndrome. Indeed, ATR inhibition induced elevated levels of binucleate cells, suggesting an important role for ATR in maintenance of genome stability during transit through late cytokinesis and abscission.

Mechanistically, we identify a role for CDC7 and the ESCRT-III subunit, CHMP4C, as upstream regulators of ATR’s localization to the midbody of late cytokinetic cells (see model in Figure S5E). Once at the midbody, ATR negatively regulates the recruitment of CHMP4B, the key effector of abscission, thereby ensuring normal abscission. In addition to negatively regulating the assembly of this effector of abscission, ATR also positively regulates the localization of a known inhibitor of abscission, ANCHR. Intriguingly, localization of ATR appears to be dependent upon ETAA1, a known activator of ATR, while being TOPBP1 independent. However, we cannot exclude the possibility that the reduction of ATR-T1089p at the midbody upon ETAA1 depletion is confounded by prior ETAA1-dependent defects in mitosis. This is consistent with TOPBP1 largely functioning during S phase while ETAA1 is more important during mitosis (Bass and Cortez, 2019). Thus, through the coordinated regulation of both positive and negative regulators of abscission ATR, potentially with one of its activators, ETAA1 ensures the correct timing of abscission which in turn contributes to genome stability.

Our work highlights a non-canonical pathway for ATR in the final stages of the cell cycle that is distinct from that employed during replication stress and DNA damage responses. An ssDNA and RPA-independent role for ATR at the nuclear envelope in response to mechanical forces has been reported (Kumar et al., 2014). More recently, suppression of necrotic cell death caused by Ca^2+^ overload associated with ischemia has also been reported as another non-canonical and CHK1-independent function for ATR (Li et al., 2021). It is possible that further replication stress and DNA damage-independent functions for ATR remain to be discovered. Importantly, precise regulation of the final separation of cells at abscission is required for safeguarding genome integrity, likely contributing to ATR’s tumor suppressive functions.

### Limitation of the study

We used an antibody specific to a modified form of ATR (ATR-S1989p) to demonstrate localization of ATR to the midbody of late cytokinetic cells and supported this result with an antibody specific to ATRIP. Thus, our data support midbody localization of the ATR-ATRIP heterotetrameric complex. However, screening commercial and in-house antibodies raised against unmodified ATR failed to identify any suitable for immunofluorescence; while, a GFP-ATR fusion protein did not display sufficient signal. Thus, we have not demonstrated midbody localization of unmodified ATR. Similarly, commercially available and in-house antibodies against CDC7 were not suitable for immunofluorescence studies. Future work will be required to define precisely how CDC7 kinase regulates ATR midbody localization and, once there, the specific phosphorylation events required to delay the onset of abscission.

## STAR★Methods

### Key resources table

**Table:** 

REAGENT or RESOURCE	SOURCE	IDENTIFIER
**Antibodies**		

Rabbit-polyclonal anti-phospho-ATR-T1989p	GenTex	#GTX128145; RRID: AB_2687562
Mouse-monoclonal anti-α-tubulin (Clone B512)	Sigma-Aldrich	#T5168; RRID: AB_477579
Mouse-monoclonal anti-AIM-1 (AuroraB)	BD Biosciences	#611082; RRID: AB_2227708
Rabbit-polyclonal anti-CHMP4B	ProteinTech	#13683-1-AP; RRID: AB_2877971
Rabbit-polyclonal anti-ATRIP	Bethyl	#A300-095A; RRID: AB_242514
Rabbit-polyclonal anti-phospho-RPA32-S33p	Bethyl	#A300-246A; RRID: AB_2180847
Mouse-monoclonal anti-ATR (C-1)	SantaCruz Biotechnology	#sc-515173; RRID: AB_2893291
Mouse-monoclonal anti-SC35	Abcam	#ab11826; RRID: AB_298608
Mouse-monoclonal anti-PML	Abcam	#ab96051; RRID: AB_10679887
Mouse-monoclonal anti-Coilin	Abcam	# ab11822; RRID: AB_2081428
Mouse-monoclonal anti-CENPA	Abcam	# ab13939; RRID: AB_300766
Rabbit-polyclonal anti-RPA70 (RPA1)	Bethyl	# A300-241A; RRID: AB_2180681
Mouse-monoclonal anti-γ-tubulin	Sigma-Aldrich	#T6557; RRID: AB_477584
Mouse-monoclonal anti-LAP2β	BD Biosciences	# 611000; RRID: AB_398313
Rabbit-polyclonal anti-ATRIP	Millipore	# 07-625; RRID: AB_310761
Rabbit-polyclonal anti-Actin	Sigma-Aldrich	# A2066; RRID: AB_476693
Mouse-monoclonal anti-RPA, clone RPA34-19	Millipore	# MABE286; RRID: AB_11203661
Mouse-monoclonal anti-TOPBP1	BD Biosciences	# 611875; RRID: AB_399355
Rabbit-polyclonal anti-ETAA1	Novus Biologicals	#NBP1-90473; RRID: AB_11012531
Rabbit-polyclonal anti-ZFYVE19 (ANCHR)	Bethyl	# A301-808A; RRID: AB_1233064
Rabbit-polyclonal anti-MKLP1 (N-19)	SantaCruz Biotechnology	# sc-867; RRID :AB_631959
Rabbit-polyclonal anti-NUP153	Bethyl	# A301-788A; RRID: AB_1211259
Rabbit-polyclonal anti-CHK1 (FL-476)	SantaCruz Biotechnology	# sc-7898; RRID: AB_2229488
Rabbit-polyclonal anti-phospho-CHK1-S317p (D12H3)	Cell Signaling Technology	# 12302; RRID: AB_2783865
Rabbit-polyclonal anti-phospho-MCM2-S40/41p	Fitzgerald et al. (2014)	N/A
Rabbit-polyclonal anti-CHMP4C	Abcam	ab155668; RRID: AB_2892638
Goat anti-Mouse IgG-heavy and light chain cross-adsorbed Antibody DyLight® 550	Bethyl	# A90-516D3; RRID: AB_10634387
Goat anti-Rabbit IgG-heavy and light chain cross-adsorbed Antibody FITC Conjugated	Bethyl	# A120-201F; RRID: AB_67263
Goat anti-Mouse IgG (H + L) Secondary Antibody, HRP	ThermoFisher	# 31430; RRID: AB_228307
Peroxidase-AffiniPure Goat Anti-Rabbit IgG (H + L)	Jackson ImmunoResearch	# 111-035-144; RRID: AB_2307391

**Bacterial and virus strains**		

One Shot™ TOP10 Chemically Competent *E. coli*	Invitrogen	#C404010

**Chemicals, peptides, and recombinant proteins**		

ATRi (ETP-46464)	SelleckChem	#S8050
ATRi (VE821)	SelleckChem	#S8007
ATMi (KU55933)	SelleckChem	#S1092
DNA-PKi (NU7026)	SelleckChem	#S2893
CHK1i (MK-8776)	SelleckChem	#S2735
CDC7i (XL413)	SelleckChem	#S7547
CDC7i (PHA-767491)	SelleckChem	#S2742
AuroraBi (AZD1152)	SelleckChem	#S1147
Benzonase	Sigma	#E1014
Paraformaldehyde	EMS	#15710
VectraShield with DAPI	VectorLabs	#H-1200
Lambda Protein Phosphatase (Lambda PP)	NEB	#P0753S

**Deposited data**		

Raw and analyzed data	This paper	This paper

**Experimental models: Cell lines**		

HeLa	ATCC	#CCL-2
HeLa H2B-GFP/mCherry-tubulin	Kaczmarczyk and Sullivan (2014)	N/A
hTERT-RPE1	ATCC	#CRL-4000

**Oligonucleotides**		

Silencer™ Negative Control No. 1 siRNA	Ambion	# AM4611
siLuciferase GL2: seq: 5′-CGUACGCGGAAUACUUCGA-3′	Eurofins	N/A
siATR; seq: 5′-CCUCCGUGAUGUUGCUUGA-3′	Eurofins	N/A
siATRIP; seq: 5′-AAGGUCCACAGAUUAUUAGAU-3′	Eurofins	N/A
siRPA2; seq: 5′-GCACCUUCUCAAGCCGAAA-3′	Eurofins	N/A
siRPA1; seq: 5′-AACUGGUUGACGAAAGUGGUG-3′	Eurofins	N/A
siTOPBP1	Dharmacon	# L-012358-00-0005
siETAA1; seq: 5′-UGACAAAGCAGUUAGGUAA-3′	Eurofins	N/A
siNUP153	Dharmacon	# L-005283-00-0005
siCHMP4B	Dharmacon	# L-018075-01-0010
siCHMP4C	Dharmacon	# L-015932-01-0010

**Software and algorithms**		

ImageJ	Schindelin et al. (2012)	https://ImageJ.nih.gov/ij/
Deltavision SoftWoRx	SoftWoRx	

### Resource availability

#### Lead contact

Further information and requests for resources and reagents should be directed to and will be fulfilled by the lead contact, Noel Francis Lowndes (noel.lowndes@nuigalway.ie).

#### Materials availabililty

All unique reagents and biological material generated in this study are available from the lead contact, Noel Francis Lowndes.

### Experimental model and subject details

#### Cell culture

HeLa cells (ECACC 93021013) were cultured in DMEM (Sigma) supplemented with 10% FBS (Gibco) and 1% penicillin/streptomycin (Sigma) at 37°C/5% CO_2_. Non-Kyoto HeLa cells stably expressing mCherry-Tubulin and GFP-H2B were a kind gift from Prof. Kevin Sullivan (Centre for Chromosome Biology, NUI Galway). hTERT-RPE1 cells were cultured in DMEM-F12 (Sigma) supplemented with 10% FBS (Gibco) and 1% penicillin/streptomycin (Sigma) at 37°C/5% CO_2_. Cells were routinely tested for mycoplasma contamination every 8 weeks.

hTERT-RPE1 cells were verified by STR analysis (Eurofins Genomics). All cell lines used are female.

### Method details

#### Cell transfection and drug treatments

For siRNA transfections, cells were transfected with Oligofectamine Reagent (LifeTechnologies) according to manufacturer’s instructions. In brief, 1.5 × 10^5^ cells were plated on a 35mm cell culture dish 24h prior to transfection. Cells were transfected with 40pmol of negative control siRNA (Dharmacon) or siRNA targeting the gene of interest (siRNA sequences are stated in the key resources table). 3h after transfection, 3X media (Media supplemented with 20% FBS and 4mM L-Glutamine (Sigma) was added to the cells and a further 1mL of media was added 24h later. Cells were harvested 48h post transfection.

Inhibitors used in this study are as follows: ATRi (ETP-46464 and VE-821, both at 10μM), ATMi (KU55933, 10μM), Aurora Bi (AZD1152, 1μM), CDC7 (PHA-767491 and XL413, both at 10μM), DNA-PKi (Nu7026, 10μM). All inhibitors were purchased from SelleckChem.

#### Immunofluorescence

Coverslips were sterilised in the cell culture dish under UV prior to addition to cells. Cells were grown on a coverslip to 70% confluency and, after indicated treatments, fixed with either 4% PFA (EMS) for 10 min at RT before permeabilisation with 0.25% Triton X-100 in PBS for 2 min at RT or with 100% MeOH for 3 min at RT depending on the antibody used. Cells were incubated with blocking solution (1% BSA in 1xPBS) for 30min, then incubated with primary antibodies (diluted in blocking solution) for 1h at 37°C or overnight at 4°C and subsequent secondary antibody for 45 min at 37°C, both in a humidified environment. Cells were washed twice in PBS between stainings and rinsed in MilliQ water before being mounted on a microscope slide using Vectashield mounting media with DAPI (VectorLaboratories, H1200).

Images were acquired on a DeltaVision integrated microscope system using the Applied Precision SoftWoRx acquisition software mounted on an IX71 Olympus microscope with a UPLFLN 40x or 100× oil objective (numerical aperture [NA] 1.3) (Imsol). All images were taken as Z-slices (0.2–0.5μm thickness) using a CoolSNAP HQ2 ICX-285 CCD camera. Parameters for image acquisition were kept constant throughout each experiment.

For analysis of protein localisation to the midbody, only late stage cytokinetic cells were included in the analysis. Early cytokinesis was defined as the stage in which: i) a cytoplasmic canal has formed with characteristic microtubule bundles and ii) the chromatin in the nuclei of the separating daughter cells is not fully decondensed. Late cytokinetic cells were defined as those in which the cytoplasmic canal is fully formed and the nuclear chromatin is decondensed.

Images were deconvolved using SoftWoRx conservative deconvolution. Quantification was carried out using FIJI software (Schindelin et al., 2012) on ‘Sum Slices’ projected images of the same depth. For representative images, the slices were projected using ‘Max Projection’.

#### Time-lapse imaging

HeLa cells stably expressing GFP-H2B and mCherry-Tubulin were grown in DMEM (Sigma) on μ-Slide 4 well glass bottom plates (ibidi). Immediately prior to imaging, cells were treated with 10μM ATRi or mock (DMSO) diluted in DMEM media (no phenol red) supplemented with 25mM HEPES pH 7.5 (Gibco), 10% FBS (Gibco) and 1% penicillin/streptomycin (Sigma). Images were acquired every 10min for 18h on a DeltaVision Elite integrated microscope system using the Applied Precision SoftWoRx acquisition software mounted on an IX71 Olympus microscope with a UPLFLN 40× oil objective (numerical aperture [NA] 1.3) (Imsol). Imaging was performed using A CoolSNAP HQ2 ICX-285 CCD camera in a chamber maintained at 37°C and supplied with 5% CO_2_ (Precision Control). Mitotic timing was defined as the time taken from prophase (chromosome condensation) to telophase (pinching of cellular membrane, condensed chromosomes). Abscission timing was determined as time taken from late telophase/early cytokinesis (formation of the cytoplasmic canal) to severing of the microtubules in the cytoplasmic canal (completed cell separation).

#### Mitotic and cytokinetic profiling

HeLa cells were grown on coverslips and transfected with siRNA as described above or treated with inhibitors as described in the figure legends. Cells were then fixed in 4% PFA in PBS for 10 min at RT and permeabilised with 0.25% Triton in PBS for 2 min at RT. Cells were then blocked for 30min in 1% BSA in PBS, followed by two PBS washes. Cells were stained for tubulin (1:500 dilution) and ANCHR (1:200 dilution) in Blocking solution (1% BSA in PBS) for 1h at 37°C in a humidified chamber, washed and stained with secondary antibodies (1:400 dilution) for 45 min at 37°C in a humidified chamber. Cells were then washed twice in PBS and rinsed in MilliQ water before being mounted on a microscope slide using Vectashield mounting media with DAPI (VectorLaboratories, H1200).

Images were aquired on a DeltaVision integrated microscope system using the Applied Precision SoftWoRx acquisition software mounted on an IX71 Olympus microscope with a UPLFLN 40x oil objective (numerical aperture [NA] 1.3) (Imsol). Images were taken using a CoolSNAP HQ2 ICX-285 CCD camera. An automatic 8 × 8 grid at 0.5μm spaced Z-Slices with a total 8μm thickness was used to avoid bias during field selection.

Quantification of cells in mitosis and cytokinesis was performed manually. DAPI was used to determine the stage of mitosis and only cells positive for α-Tubulin at the cytoplasmic canal and ANCHR at the midbody were scored for cytokinesis.

#### SDS-PAGE and western blotting

Cells were lysed in lysis buffer (50mM Tris-HCL pH 7.5, 150mM NaCl, 0.5% NP-40, 10% glycerol, 1x protease and phosphatase inhibitors) on ice for 1h with vortexing at 15min intervals. Cleared lysates were collected after centrifugation at 14,000rpm for 15mins, separated by SDS-PAGE and then transferred onto a nitrocellulose membrane. Membranes were incubated in blocking solution (5% milk in 1x TBS +0.1% Tween 20) followed by overnight incubation in primary antibodies diluted in blocking solution or 1% BSA (bovine serum albumin) in 1x TBS +0.1% Tween 20.

### Quantification and statistical analysis

Statistical details of each experiment can be found in the Figure Legends. The number of experimental replicates is indicated in the figures or Figure Legends. For analysis of live cell imaging random fields with ≥5 cells were selected. Experiments were designed to ensure conformity with standards reported in other publications. All statistical analysis was performed on Graph Pad Prism (GraphPad version 9). Statistical significance is indicated in the Figure Legends and data presented in graphs are mean and SD unless stated otherwise.

## Data Availability

All data produced in this study are included in the published article and its supplementary information, or are available from the lead contact upon request. This paper does not report original code. Any additional information required to reanalyze the data reported in this paper is available from the lead contact upon request.
