# Supplementary material for: A function for ataxia telangiectasia and Rad3-related (ATR) kinase in cytokinetic abscission
Source: iScience. 2022 Jun 6;25(7):104536. doi: 10.1016/j.isci.2022.104536 (PMC9213759; doi:10.1016/j.isci.2022.104536)
Supplement: Document S1. Figures S1–S5 [file mmc1.pdf]

iScience, Volume *25*

Supplemental information

A function for ataxia telangiectasia and Rad3-related (ATR) kinase in cytokinetic abscission

Janna Luessing, Chituru C. Okowa, Emer Brennan, Muriel Voisin, and Noel F. Lowndes

Supplemental Material

Supplemental Figures:

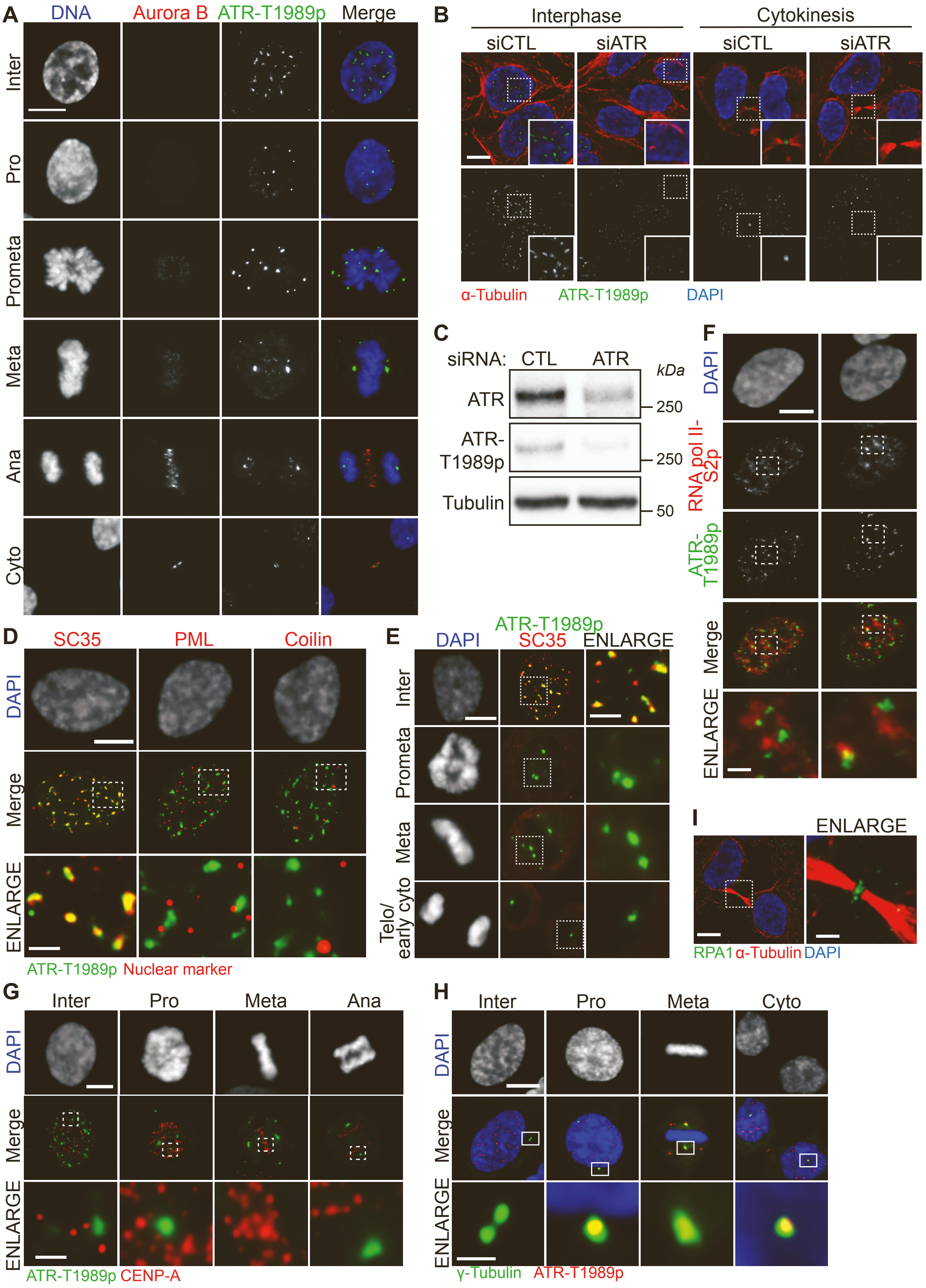

Supplementary Figure 1. Localisation of ATR-T1989p is detectable during interphase and mitosis independent of DNA and RPA2 but dependent upon ATRIP. (Related to Figure 1) **(A)** ATR-T1989p and Aurora B localisation during interphase and mitosis in asynchronous hTERT-RPE1 cells. **(B)** Localisation of ATR-T1989p in interphase (left panel) and to the midbody (right panel) upon ATR depletion in HeLa cells. **(C)** Western blot confirming the extent of ATR depletion in (B). **(D)** Co-localisation of ATR-T1989p and the nuclear markers, SC35 (nuclear speckles), PML bodies and Coilin (Cajal bodies) during interphase. **(E)** Localisation of ATR-T1989p and SC35 during mitosis and co-localisation in interphase. **(F)** Localisation ATR-T1989p and RNA pol II-S2p during interphase. **(G)** Localisation of ATR-T1989p and CENPA during mitosis. **(H)** Co-localisation of ATR-T1989p and γ-Tubulin at centrosomes during mitosis. **(I)** Localisation of RPA1 during cytokinesis. All scale bars are 10μm, all enlarged image scale bars are 2μm.

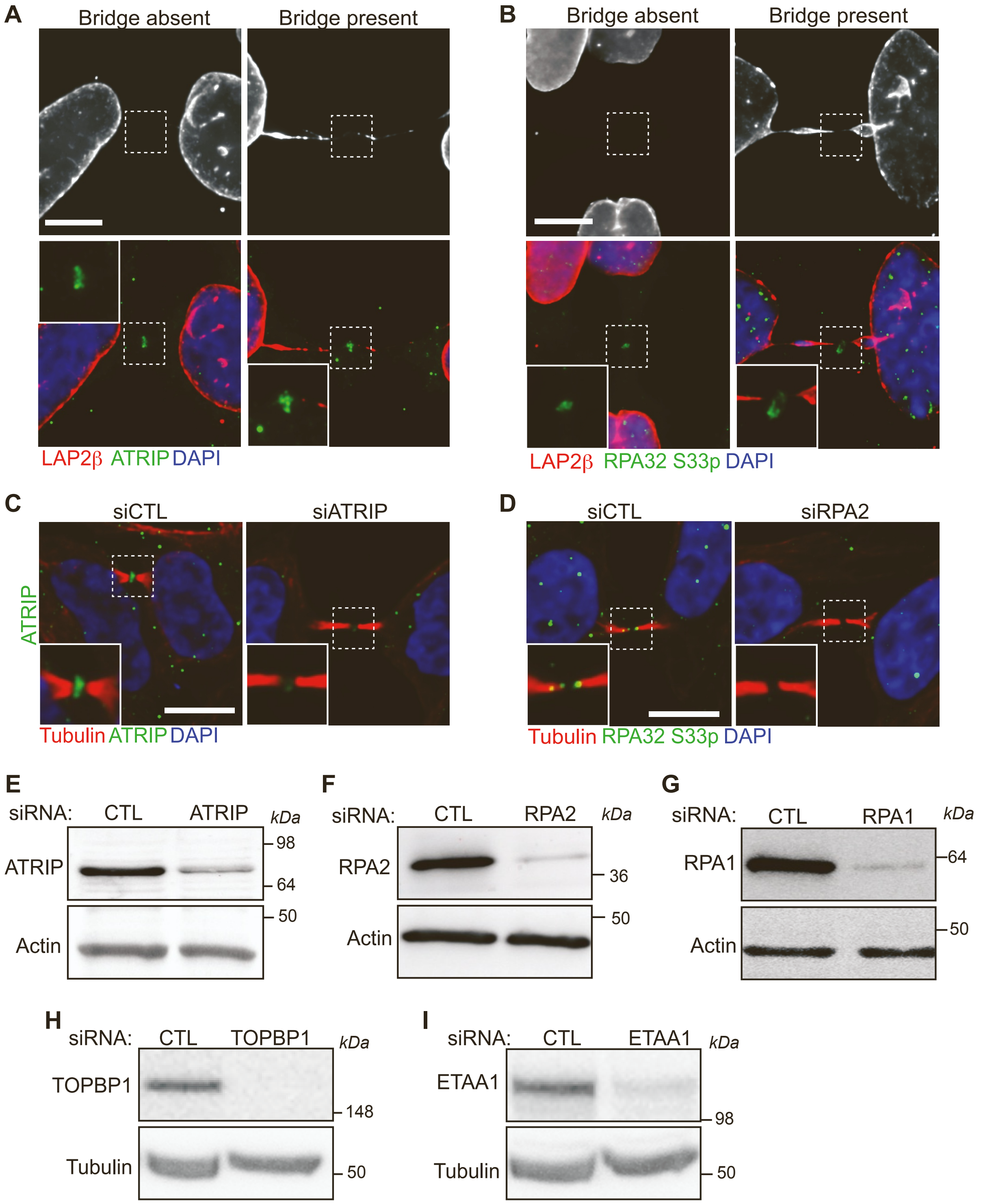

Supplementary Figure 2. Localisation of ATR-T1989p is detectable during interphase and mitosis independent of DNA and RPA2 but dependent upon ATRIP. (Related to Figure 2) **(A)** Localisation of ATRIP to midbodies of all late cytokinetic cells irrespective of the presence of absence of LAP2β bridges. **(B)** Localisation of RPA2-S33p to midbodies of all late cytokinetic cells irrespective of the

presence of absence of LAP2β bridges. **(C)** Localisation of ATRIP to the midbody upon ATRIP depletion in HeLa cells. **(D)** Localisation of RPA2-S33p to the midbody upon RPA2 depletion in HeLa cells establishes the specificity of the RPA2-S33p antibody. **(E)** Western blot confirming the extent of ATRIP depletion. **(F)** Western blot confirming the extent of RPA2 depletion. **(G)** Western blot confirming the extent of RPA1 depletion. **(H)** Western blot confirming the extent of TOPBP1 depletion. **(I)** Western blot confirming the extent of ETAA1 depletion. All scale bars are 10μm. HeLa cells used in A-I.

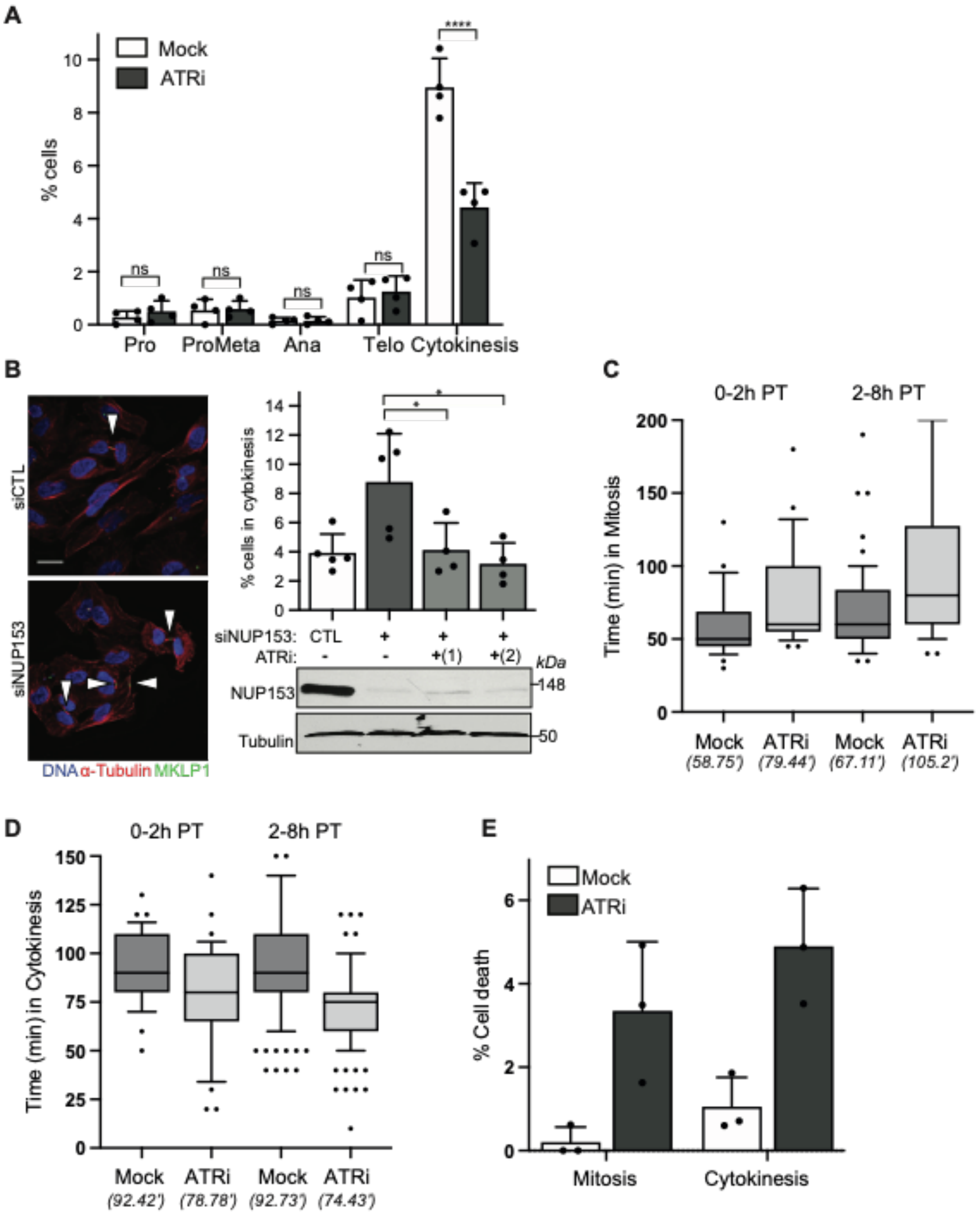

Supplementary Figure 3. Mitotic and cytokinetic analysis of ATR inhibited cells. (related to Figure 3) **(A)** Percentage of Hela cells in distinct phases of mitosis and cytokinesis upon ATR inhibition (ETP-46464, 10 μM, 1h). At least 400 cells were analysed in each of three independent experiments (****p < 0.0001, Student's t-test). **(B)** Representative image of control (siCTL) or NUP153 depleted (siNUP153) cytokinetic cells. Quantification of cells in cytokinesis after NUP153 depletion. Cells

were treated with control or NUP153 siRNA for 48hrs followed by 3hr ATR inhibition as indicated (10μM, ATRi (1) = ETP-46464 and ATR = VE-821 as indicated). Cell lysates were analysed by western blotting to demonstrate the extent of NUP153 depletion. Cytokinetic cells, indicated by arrowheads, were scored manually. >200 cells were analysed in each of four experiment (*p=0.04, Student's t-test). **(C)** Mitotic timing of cells either mock or ATR inhibitor (ETP-46464, 1h, continuous) treated. Samples are divided into two distinct sub-groups: cells entering mitosis during short ATR exposure within the first 2h (0-2PT) and cells entering mitosis during longer ATR exposure after 2h (2-8h PT). Quantification is across three independent experiments Box plot of panels indicate the median and quartile ranges. **(D)** Cytokinetic timing of cells either mock or ATR inhibitor (ETP-46464) treated. Samples are divided into two distinct sub-groups: cells entering mitosis during short ATR exposure within the first 2h (0-2PT) and cells entering mitosis during longer ATR exposure after 2h (2-8h PT). Quantification is across three independent experiments. Box plot of panels indicate the median and quartile ranges. **(E)** Percentage of cell death in mitosis and cytokinesis upon ATR inhibition (ETP-46464, 10μM, continuous). Cell death was measured as cells undergoing nuclear fragmentation. Dot plots indicate the average of three independent experiments (*$p=0.0199$; **$p=0.0069$, Student's t-test). Data are represented as mean ±SD of the individual replicates. All scale bars are 10μm. HeLa cells used in A-G.

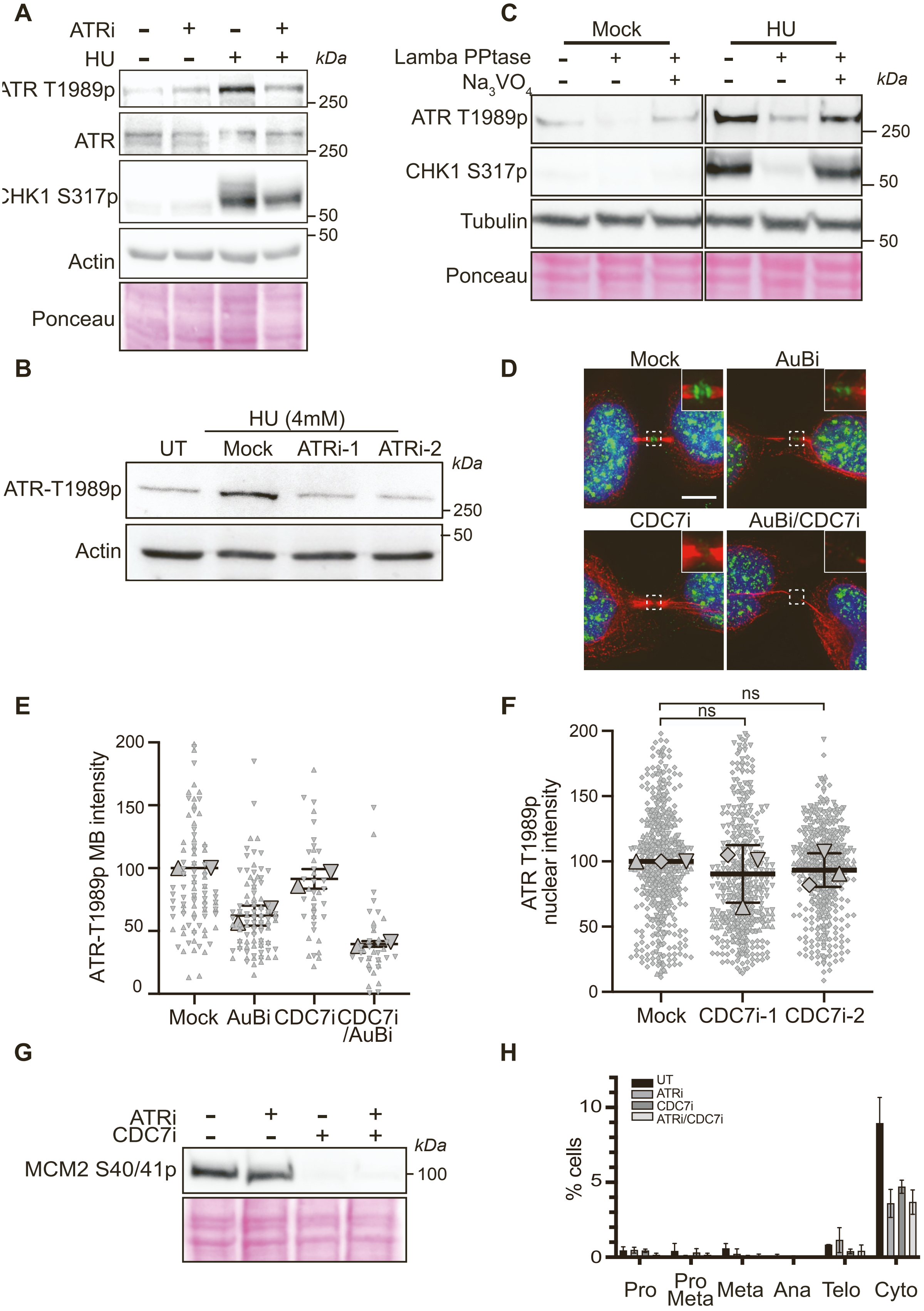

A
ATRi
HU
kDa
ATR T1989p
ATR
CHK1 S317p
Actin
Ponceau
250
250
50
50
B
HU (4mM)
UT
Mock
ATRi-1
ATRi-2
kDa
ATR-T1989p
Actin
250
50
C
Mock
HU
Lamba PPtase
Na3VO4
kDa
ATR T1989p
CHK1 S317p
Tubulin
Ponceau
250
50
50
D
Mock
AuBi
CDC7i
AuBi/CDC7i
E
ATR-T1989p MB intensity
200
150
100
50
0
Mock
AuBi
CDC7i
CDC7i /AuBi
F
ns
ns
ATR T1989p nuclear intensity
200
150
100
50
0
Mock
CDC7i-1
CDC7i-2
G
ATRi
CDC7i
kDa
MCM2 S40/41p
100
H
UT
ATRi
CDC7i
ATRi/CDC7i
% cells
10
5
0
Pro
Pro Meta
Meta
Ana
Telo
Cyto

Supplementary Figure 4. Analysis of ATR-T1989p upon ATR, Aurora B and CDC7 inhibition. (Related to Figure 4) **(A)** Western blot of ATR-T1989p upon treatment with ATR inhibitor (ATRi-1= ETP-46464, 10μM, 1h) followed by addition of HU (4mM HU, 4h) in the continuous presence of the ATR inhibitors. **(B)** Western blot of ATR-T1989p upon treatment with ATR inhibitors (ATRi-1= ETP-46464, ATRi-2 = VE-821, 10μM, 1h) followed by addition of HU (4mM HU for 4h) in the continuous presence of the ATR inhibitors **(C)** Lambda protein phosphatase analysis of ATR-T1989p and CHK1 phosphorylation. Tubulin is used as a loading control. **(D)** Localisation of ATR-T1989p to the midbody of late cytokinetic cells in response to Aurora B (AZD1152, 1 μM, 1h) and/or CDC7 (XL413, 10 μM, 1h) inhibition. **(E)** Quantification of ATR-T1989p intensity at late cytokinetic midbodies upon inhibition of Aurora B (AZD1152, 1 μM, 1h) and/or CDC7 (XL413, 10 μM, 1h). **(F)** Quantification of ATR-T1989p nuclear intensity upon inhibition of CDC7 using two chemically distinct inhibitors (CDC7i-1 = XL413, 10 μM, 1h; CDC7i-2 = PHA-767491, 10 μM, 1h). (ns, Student's t-test). Errors bars indicate SD. **(G)** Western blot of MCM2-S40/41p validating CDC7 (XL413, 10 μM, 1h) inhibition. **(H)** Percentage of cells in distinct phases of mitosis and cytokinesis upon ATR (ETP-46464, 10 μM, 1h) and/or CDC7 (XL413, 10 μM, 1h) inhibition. At least 400 cells were analysed in each of three independent experiments. Data are represented as mean ±SD of the individual replicates All scale bars are 10μm. HeLa cells used in A-H

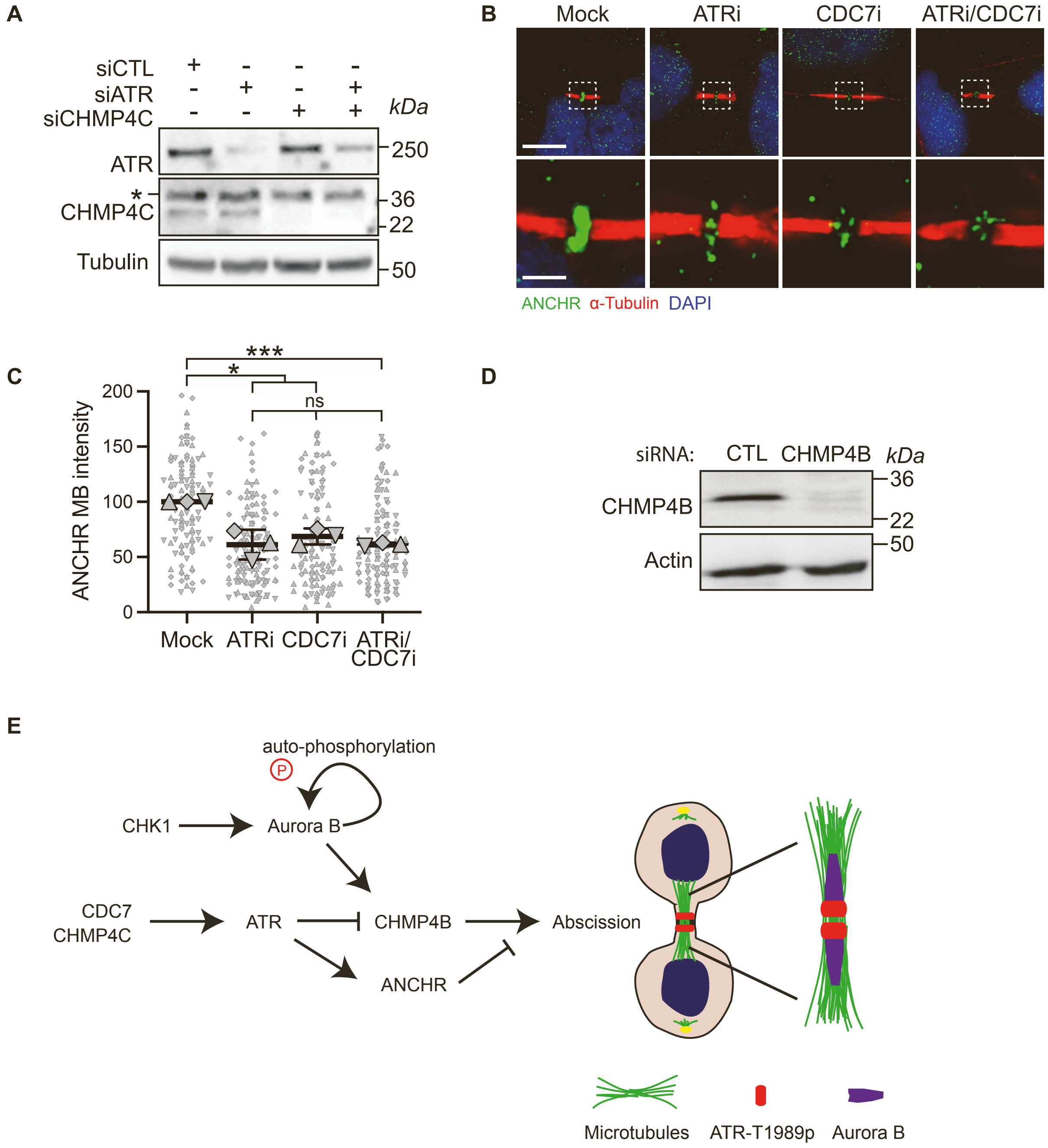

Supplementary Figure 5. ATR and CDC7 are required for recruitment of ANCHR to midbody, but only ATR is required for the regulation of CHMP4B. (Related to Figure 5) **(A)** Western blots demonstrating the extent of ATR and CHMP4C depletion. **(B)** Localisation of ANCHR to the midbody upon inhibition of CDC7 and/or ATR (CDC7i-1 = XL413, 10 µM and ATRi = ETP46464, 10µM, 1h). **(C)** Quantification of ANCHR intensity upon inhibition of CDC7 (CDC7i-1 = XL413, 10 mM, 1h) and or ATR (ETP-46464, 10 µM, 1h). (**p=0.038 (ATRi), *p=0.0174 (CDC7i)*, ****p=0.0005 (ATRi/CDC7i)*, Student's t-test). Data are represented as mean ±SD of the individual

replicates. All scale bars are 10μm, all enlarged image scale bars are 2μm. **(D)** Western blots demonstrating the extent of CHMP4B depletion. **(G)** Model of the ATR-regulated pathway controlling abscission. Aurora B separately regulates abscission upstream of CHMP4B (Steigemann et al 2009).
